# ENO1 as an Immunoregulatory Hub in Cancer: Mechanisms and Translational Implications

**DOI:** 10.3390/biom16071050

**Published:** 2026-07-18

**Authors:** Giovanni Perconti, Angela Bonura, Patrizia Rubino, Agata Giallongo

**Affiliations:** 1 Institute of Translational Pharmacology (IFT), National Research Council (CNR), Via Ugo La Malfa 153, 90146 Palermo, Italy; angela.bonura@ift.cnr.it (A.B.); patrizia.rubino@ift.cnr.it (P.R.); 2 Institute of Biomedical Research and Innovation (IRIB), National Research Council (CNR), Via Ugo La Malfa 153, 90146 Palermo, Italy; agata.giallongo@irib.cnr.it

**Keywords:** ENO1, alpha-enolase, tumor-associated antigen, tumor microenvironment, citrullination, myeloid cells, cancer immunotherapy, immune escape

## Abstract

Alpha-enolase (ENO1) is a multifunctional protein frequently overexpressed in solid tumors, where elevated levels are associated with aggressive behavior and poor prognosis. Beyond its canonical glycolytic role, ENO1 participates in immunoregulatory processes through distinct subcellular pools. Intracellular ENO1 shapes tumor-associated metabolic programs, while surface-exposed ENO1 functions as a plasminogen receptor and can engage innate immune signaling pathways. Post-translational modifications—particularly citrullination and phosphorylation—generate structurally altered epitopes that expand ENO1 antigenicity and enable adaptive immune recognition, including coordinated humoral and T-cell responses in cancer patients. These determinants of ENO1 immunogenicity have downstream consequences within the tumor microenvironment: immune-accessible ENO1 modulates myeloid cell recruitment, dendritic cell maturation, and macrophage polarization, while ENO1-dependent metabolic and signaling programs contribute to immune suppression and escape through multiple interconnected axes. Together, these mechanisms position ENO1 at the interface between tumor metabolism and immune regulation. Preclinical evidence demonstrates that ENO1-directed strategies—including antibody-based targeting, DNA vaccination, and vaccines incorporating post-translationally modified ENO1 peptides—can generate productive antitumor immunity and synergize with checkpoint blockade, supporting the rationale for ENO1 as an immunotherapeutic target. This review synthesizes current evidence within an integrated framework linking ENO1 dysregulation to its immunological consequences in cancer and discusses translational implications for ENO1-centered immunotherapy and immunoprevention.

## 1. Introduction

Alpha-enolase (ENO1) is a highly conserved and ubiquitously expressed protein that catalyzes the conversion of 2-phosphoglycerate to phosphoenolpyruvate in the glycolytic pathway [1,2]. Although originally characterized as a constitutive metabolic enzyme, ENO1 is now recognized as a multifunctional protein whose biological outputs depend on cellular context and subcellular localization [1,2]. The *ENO1* gene gives rise not only to the cytosolic glycolytic enzyme but also to c-Myc promoter-binding protein 1 (MBP-1), a nuclear isoform generated through alternative translation initiation [3,4]. MBP-1 was initially identified for its ability to repress *c-MYC* transcription and was subsequently shown to regulate additional cancer-relevant genes, including *ERBB2* and *COX-2*, thereby linking *ENO1* gene products to transcriptional programs controlling proliferation and inflammation [4,5,6].

In addition to the ENO1/MBP-1 axis, ENO1 has long been recognized as a moonlighting protein involved in diverse non-canonical intracellular processes that extend beyond glycolysis. These include roles in cytoskeletal dynamics and migratory behavior, stress-response and survival programs, and broader protein–protein interaction networks, supporting the view that ENO1 participates in context-dependent regulatory programs not strictly confined to metabolic flux [1,7]. More recently, ENO1 has also been identified as a bona fide RNA-binding protein capable of interacting with large sets of cellular RNAs. These interactions can modulate ENO1 enzymatic activity through a mechanism termed riboregulation and are dynamically regulated by post-translational modifications such as lysine acetylation (Table 1), linking ENO1 to RNA-dependent regulatory circuits that influence cellular metabolism and differentiation [8].

A further dimension of ENO1 multifunctionality is its capacity to associate with the plasma membrane. ENO1 has been reported at the cell surface of diverse cell types, where it can function as a plasminogen receptor and promote localized proteolytic activity [9,10]. This membrane-associated role contributes to pericellular matrix remodeling and cell migration, processes that are relevant to inflammatory cell recruitment and are also physiologically important for tissue remodeling and wound repair [11]. In pathological settings such as cancer, dysregulated ENO1 expression and altered regulatory control can amplify and exploit these pre-existing activities, increasing their impact on tissue remodeling, motility programs, and disease progression [12,13]. Consistent with these properties, altered ENO1 expression is a recurrent feature of malignant transformation, particularly in solid tumors, where elevated ENO1 levels are frequently associated with aggressive behavior, therapy resistance, and unfavorable clinical outcomes [12,13,14].

In parallel with its cell-intrinsic roles, ENO1 has substantial immunological relevance. ENO1 is a recognized tumor-associated antigen and can elicit both humoral and cellular immune responses in cancer patients, with immunogenicity influenced by factors such as protein abundance, subcellular localization, and post-translational modifications (PTMs) [13,15,16]. Among these determinants, covalent protein modifications are increasingly recognized as relevant contributors to ENO1 immunogenicity. These modifications can alter structural and functional properties of the protein, potentially influencing its interaction with the immune system. Consideration of these molecular features is therefore important to understand the conditions under which ENO1 becomes an immune target in cancer and inflammatory diseases.

Notably, ENO1 exposure is not restricted to malignant cells but can also occur in immune-cell populations under conditions of stress or activation. These contexts can promote the appearance of ENO1 at the cell surface, increasing its immunological accessibility in inflammatory environments, including conditions associated with immune-cell turnover and apoptosis [17].

Tumor progression is critically shaped by interactions with the host immune system. Through immunoediting and microenvironmental remodeling, tumors evolve under immune pressure while actively suppressing effective antitumor responses [18,19]. Metabolic constraints, cytokine networks, stromal architecture, and immune-cell recruitment and polarization collectively determine whether immune surveillance is maintained or bypassed [20,21]. Proteins that integrate metabolic activity with extracellular interactions and antigenic potential are therefore well positioned to influence tumor–immune crosstalk [22,23]. In this context, ENO1 emerges as a compelling candidate, as its deregulation in cancer intersects with pathways governing metabolism, migration, proteolysis, and immune recognition. Notably, ENO1-directed immunity may also have clinical relevance, as spontaneous immune responses against ENO1 have been associated with indicators of improved disease control in cancer patients [24]. 

**Figure 1:**
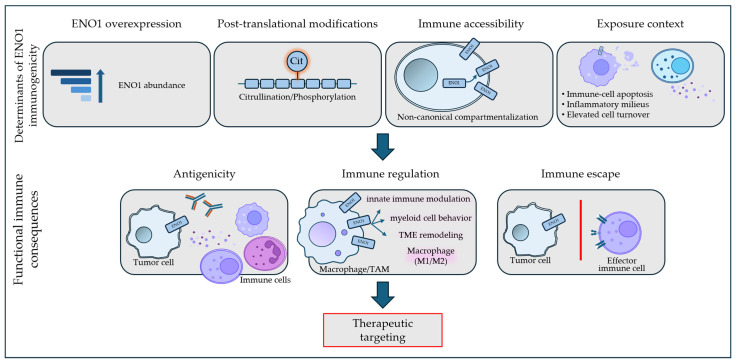
Conceptual framework of ENO1 immunogenicity in cancer. The upper panel depicts the main classes of determinants that contribute to ENO1 immune visibility, encompassing quantitative (ENO1 overexpression), qualitative (post-translational modifications), spatial (immune accessibility), and contextual factors (exposure context, including inflammatory conditions, cell turnover, and immune-cell apoptosis). These features collectively enhance ENO1 antigenicity and accessibility. The central panel illustrates the downstream immunological consequences within the tumor microenvironment, including modulation of innate and adaptive immune responses and the establishment of regulatory and immune-escape mechanisms. The lower panel indicates the conceptual link to therapeutic targeting of ENO1-associated pathways. Abbreviations: TME, tumor microenvironment; TAM, tumor-associated macrophage; M1/M2, macrophage polarization states.

As schematically illustrated in Figure 1, this review is organized around a progressive framework linking ENO1 dysregulation to its immunological consequences in cancer. For clarity, the mechanisms discussed in this review are organized into three conceptual layers: (i) determinants of ENO1 immunogenicity (Section 2 and Section 3), which define antigen availability and visibility; (ii) functional immune effects (Section 4), describing how immune-accessible ENO1 modulates innate and adaptive responses; and (iii) immune suppression and escape mechanisms (Section 5), where these processes converge into defined immunosuppressive outputs. Finally, these interconnected processes provide the basis for exploring therapeutic strategies targeting ENO1-associated pathways (Section 6).

Together, this integrated perspective highlights ENO1 as a multifunctional mediator linking tumor biology and immune regulation, providing a conceptual basis for understanding its context-dependent roles in tumor progression and its potential as a therapeutic target.

## 2. ENO1 as a Tumor-Associated Immunogenic Antigen in Cancer

### 2.1. ENO1 as a Tumor-Associated Antigen

Evidence accumulated over the past two decades indicates that ENO1 is a bona fide tumor-associated antigen capable of eliciting spontaneous immune responses in cancer patients. Initial serological and immunoproteomic studies in pancreatic ductal adenocarcinoma identified ENO1 among antigens recognized by patient sera and documented circulating anti-ENO1 autoantibodies, with substantially higher frequencies than in controls [25,26]. Similar observations have subsequently been reported in other solid tumors, including lung and additional epithelial cancers, supporting the view that ENO1 represents a recurrent tumor-associated antigen rather than a marker restricted to a single tumor type [12,13,15,27].

Anti-ENO1 autoantibodies can show clinically meaningful dynamics. In non-small cell lung cancer, an increase in anti-ENO1 antibody levels after tumor resection has been associated with longer disease-free survival, suggesting that these antibodies may reflect active antitumor immune surveillance [28]. Interestingly, higher titers of anti-ENO1 antibodies have also been reported to correlate with a greater frequency of ENO1-specific T cells, suggesting coordinated humoral and cellular responses against ENO1 in cancer patients [24].

These studies also indicate that tumor-associated ENO1 expression and ENO1-directed immune responses represent distinct biological readouts. In NSCLC, increased ENO1 expression in tumor tissues was associated with poorer clinical outcomes [13], whereas circulating anti-ENO1 antibodies displayed a more complex pattern: reduced antibody levels were observed in patients with advanced lung and breast cancer [15], while postoperative increases in anti-ENO1 antibodies correlated with improved progression-free survival in NSCLC [27]. Likewise, circulating ENO1-specific T cells were associated with prolonged survival in patients with pancreatic ductal adenocarcinoma [24]. Collectively, these findings suggest that ENO1 overexpression may reflect tumor aggressiveness, whereas detectable ENO1-directed immune responses may instead reflect preserved or reactivated antitumor immune surveillance.

Given that ENO1 is predominantly intracellular and lacks a canonical signal peptide, the detection of ENO1-directed adaptive immunity supports the existence of context-dependent mechanisms of antigen accessibility operating in tumor-associated microenvironments. Importantly, these accessibility mechanisms are likely driven by biological processes that are broadly shared across malignant transformation, including metabolic stress, inflammatory remodeling, and altered protein handling [13,18,19,25].

Consistent with these observations, experimental evidence demonstrates that ENO1 can elicit antigen-specific T-cell responses. ENO1-derived epitopes have been shown to be processed and presented by antigen-presenting cells, supporting activation of both helper (CD4^+^) and cytotoxic (CD8^+^) T lymphocytes in experimental models [16,29]. ENO1-specific T cells have also been detected in the peripheral blood of cancer patients, indicating that ENO1-directed responses can be systemically maintained and are not restricted to the tumor site [24]. These ENO1-specific T cells are not merely detectable but can display antigen-specific effector functions, as patient-derived clones have been shown to produce pro-inflammatory cytokines and to exert antigen-dependent cytotoxicity against target cells, supporting their potential contribution to active tumor control [24]. Importantly, ENO1-reactive T-cell immunity can emerge in vivo in tumor-bearing hosts without deliberate vaccination, indicating that ENO1 antigenicity can arise spontaneously under conditions of tumor-associated inflammation and antigen exposure [25]. Because ENO1 is a self-protein, the emergence of ENO1-directed adaptive immunity likely reflects qualitative changes in antigenic structure, including post-translational modifications that generate immunologically distinct epitopes [11,16,30].

Overall, the evidence reviewed in this section supports ENO1 as a shared tumor-associated antigen. Serological and immunoproteomic studies consistently demonstrate immune recognition of ENO1 in cancer patients [13,15,25,26,27,28], ex vivo analyses provide functional evidence for ENO1-specific T-cell responses [24], and preclinical vaccination studies show that ENO1-derived epitopes can induce protective antitumor immunity in animal models [16,29]. However, whether spontaneous ENO1-directed immune responses contribute directly to tumor control in patients remains unresolved, as the available clinical studies are primarily associative rather than mechanistic.

### 2.2. Cellular and Molecular Determinants of ENO1 Immunogenicity

The immunogenicity of ENO1 in cancer can be understood as the result of distinct but interconnected classes of determinants that collectively increase immune visibility. These include quantitative, spatial, and contextual factors, which are outlined below as a framework to organize the mechanisms discussed in subsequent sections. Quantitative factors such as overall ENO1 abundance may influence the size of the antigen pool available for processing, but current evidence suggests that qualitative and spatial alterations are more directly linked to ENO1 immunogenicity [12,31].

An important determinant is non-canonical compartmentalization and immune accessibility. In tumor and inflammatory contexts, ENO1 can become immune-accessible through increased surface display, thereby enabling antibody binding and immune capture [9,11,30]. Mechanistically, cell-surface ENO1 functions as a plasminogen receptor and supports pericellular proteolysis, thereby creating conditions that may facilitate immune exposure in inflamed tissues and tumors characterized by extensive matrix remodeling and cell turnover [9,11]. Importantly, ENO1 surface exposure can occur through regulated mechanisms rather than representing a purely passive event. In breast cancer models, STIM1/ORAI1-dependent calcium signaling has been reported to promote ENO1 translocation and non-classical externalization, with functional consequences for cell migration [32]. Additional evidence suggests that ENO1 surface exposure may be facilitated by specific protein–protein interaction networks, including Hsp70-dependent mechanisms influencing ENO1 surface localization in tumor-associated contexts [33,34].

Together with the STIM1/ORAI1- and Hsp70-dependent pathways described above, these findings indicate that ENO1 surface exposure is regulated by multiple signaling mechanisms rather than by a single conserved trafficking route.

More recently, the spectrum of mechanisms controlling ENO1 translocation to the plasma membrane has been further expanded by the identification of a post-translationally regulated pathway linking cytokine signaling to ENO1 surface exposure. In colorectal and triple-negative breast cancer models, TGF-β1 signaling was shown to activate the Smad3–PRMT5 axis, leading to symmetric dimethylation of ENO1. This modification promoted redistribution of ENO1 from the cytosol to the plasma membrane, where ENO1 engaged in non-glycolytic, membrane-associated functional interactions. Importantly, genetic or pharmacologic inhibition of PRMT5 markedly reduced ENO1 cell surface localization, demonstrating that ENO1 trafficking to the cell surface is dynamically regulated by signaling-dependent methylation [35] (Table 1).

In addition to regulated translocation pathways, tumor-cell apoptosis represents an additional mechanism that can transiently increase ENO1 accessibility at the cell surface. Accordingly, in chronic lymphocytic leukemia, ENO1 was found to translocate to the plasma membrane specifically in apoptotic leukemic cells, while remaining predominantly intracellular in viable cells, indicating that tumor-cell death can create transient windows of immune accessibility [36].

This surface accessibility is immunologically relevant because it overcomes intracellular confinement and provides a direct interface for antibody recognition, while also creating conditions that may favor antigen capture and processing in inflamed or apoptotic tissue environments [11,30].

Beyond these regulated translocation mechanisms, the broader exposure context determines whether surface-accessible ENO1 is likely to remain a local proteolytic mediator or become immunologically visible. Evidence from inflammatory models shows that cell-surface ENO1 can support plasminogen-dependent monocyte recruitment and tissue remodeling [11], whereas studies of apoptotic neutrophils and apoptotic cell membranes indicate that ENO1, including citrullinated ENO1 species, can become exposed during cell turnover [17,30]. These observations support the view that ENO1 immunogenicity may arise from the amplification of physiological exposure pathways that become exacerbated in cancer-associated inflammatory and remodeling environments.

**Figure 2:**
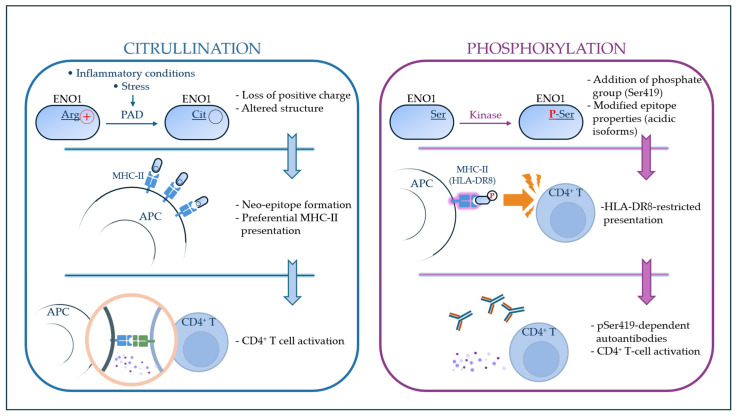
PTM-driven generation of immunologically distinct ENO1 epitopes. The diagram summarizes how citrullination and phosphorylation can modify ENO1-derived epitopes and promote immune recognition. Citrullination of arginine residues by PAD enzymes alters charge and local structure, favoring the generation of neo-self epitopes and MHC class II presentation. Phosphorylation generates phospho-ENO1 peptides, including Ser419-containing epitopes, that can be presented in an HLA-restricted manner and recognized by CD4^+^ T cells. Abbreviations: APC, antigen-presenting cell; HLA, human leukocyte antigen; MHC, major histocompatibility complex; PAD, peptidylarginine deiminase.

A complementary class of determinants is represented by qualitative modifications of the antigen itself. Consistent with the framework developed in Section 3, PTMs contribute to ENO1 antigenicity by reshaping epitope structure and immune recognition, with citrullination and phosphorylation representing the most clearly characterized modifications to date (Figure 2) [16,37].

Taken together, the evidence reviewed in this section indicates that ENO1 immune visibility is not generated by a single conserved mechanism, but by the convergence of several exposure-related processes. Regulated trafficking pathways involving calcium-dependent signaling, Hsp70-associated interactions, and PRMT5-dependent methylation have been described in selected tumor models, whereas apoptosis- and inflammation-associated exposure mechanisms have been characterized mainly in inflammatory or hematological settings [17,32,33,34,35,36]. The relative contribution of these mechanisms to ENO1 immunogenicity across tumor types remains incompletely defined and represents an important area for further investigation.

## 3. Post-Translational Determinants of ENO1 Immunogenicity

Post-translational modifications (PTMs) represent one of the mechanisms through which self-proteins can acquire novel immunological properties and become recognizable as tumor-associated antigens. This concept is increasingly recognized across multiple tumor antigens, where PTM-dependent neo-epitopes contribute to immune recognition [38,39]. By altering amino acid side chains, PTMs can change protein conformation, stability, intracellular trafficking, and, critically for immunology, the repertoire of peptides presented by major histocompatibility complex (MHC) molecules. As a result, modified self-proteins may be perceived as “neo-self” antigens, potentially favoring tolerance breakdown and the induction of adaptive immune responses.

In the case of ENO1, PTMs are emerging as relevant determinants of its immunogenicity rather than secondary features. ENO1 is highly expressed and metabolically active in tumors and chronic inflammatory environments, where inflammatory and oxidative conditions create a permissive environment for PTMs. This makes ENO1 a particularly suitable substrate for the generation of modified epitopes recognizable by B and T cells.

Although the range of ENO1 post-translational modifications is likely broader, current evidence is still limited and mainly focuses on a subset of modifications with defined immunological relevance. Two PTMs have been most clearly linked to ENO1 immunogenicity to date: citrullination and phosphorylation (Figure 2).

### 3.1. Citrullinated ENO1 as a Source of Neoantigens

Citrullination converts positively charged arginine residues into neutral citrulline, leading to physicochemical and structural alterations that can substantially affect antigen processing and peptide-MHC interactions [40,41]. In the case of ENO1, citrullinated peptides have been shown to display enhanced binding to MHC class II molecules and to elicit robust CD4^+^ T-cell responses compared with their native counterparts (Figure 2, left panel) [16]. These findings support the view that citrullination can generate structurally and immunologically altered epitopes rather than merely amplifying recognition of pre-existing self-peptides.

From an immunological standpoint, this distinction is critical. Native ENO1 is subject to central tolerance mechanisms, whereas citrullinated ENO1 peptides are expected to be less efficiently represented during thymic selection, potentially allowing the persistence of peripheral T-cell clones capable of recognizing citrullinated ENO1 epitopes with high functional avidity [40,41]. This interpretation is consistent with observations made for other citrullinated self-antigens and provides a mechanistic explanation for the strong CD4^+^ T-cell responses elicited by citrullinated ENO1 in cancer-associated settings [16,40,41]. Importantly, this mode of neoepitope generation does not require genetic mutation of ENO1 but instead reflects inflammation- and stress-driven protein editing that can render ubiquitous self-protein immunologically distinct.

The generation of citrullinated ENO1 is tightly linked to inflammatory and stress-associated environments. Peptidylarginine deiminase (PAD) enzymes, which catalyze arginine-to-citrulline conversion, are activated under conditions of elevated intracellular calcium and inflammatory signaling [42,43]. Because ENO1 is highly abundant and predominantly cytosolic, it represents a plausible substrate for PAD-mediated modification under inflammatory stress conditions. Consequently, localized citrullination of ENO1 can increase the availability of modified peptides for antigen processing and subsequent immune recognition [41].

The broader immunological relevance of ENO1 is also supported by observations in autoimmune diseases, where ENO1 and post-translationally modified self-proteins, particularly citrullinated antigens, are well-established immune targets. These findings reinforce the concept that inflammation-driven protein modification and altered antigen exposure can render ubiquitous metabolic enzymes such as ENO1 immunologically visible across distinct pathological contexts [44,45,46,47].

### 3.2. Phosphorylated ENO1

Phosphorylation represents a second, mechanistically distinct, PTM that can critically influence ENO1 immunogenicity. Unlike citrullination, which is often associated with calcium-dependent PAD enzyme activity in inflammatory or stress-related contexts, phosphorylation is a reversible modification integrated into canonical signaling pathways and cellular activation programs. Tumor cells frequently display altered kinase and phosphatase activities, leading to abnormal phosphorylation patterns that may generate immunologically relevant phospho-epitopes.

Phosphorylated ENO1 isoforms have been identified in patients with pancreatic ductal adenocarcinoma (PDAC), and autoantibodies recognizing phosphorylated ENO1 variants have subsequently been described in the same disease [26,37]. These findings indicate that phosphorylation can create antigenic determinants that are selectively targeted by the humoral immune system.

A key advance in this area is the demonstration that ENO1 phosphorylation can shape not only antibody responses but also T-cell recognition. Specific ENO1 phosphopeptides, such as those containing phosphorylated Ser419, have been predicted to bind with high affinity to selected MHC class II molecules and subsequently shown to activate CD4^+^ T cells (Figure 2, right panel) [37]. Importantly, T-cell recognition can depend on the presence of the phosphate group itself, meaning that the modified peptide is immunologically distinct from its unmodified counterpart [37].

This provides direct evidence that ENO1 phosphorylation can generate bona fide neo-epitopes rather than simply modulating antigen processing. Functionally, the immune system can distinguish between phosphorylated and non-phosphorylated ENO1, responding selectively to the modified form [37]. An additional layer of relevance comes from immunogenetics. Associations between anti-phosphorylated ENO1 responses and specific HLA class II alleles, such as HLA-DRB1*08, suggest that genetic background influences the likelihood of mounting immune responses against phospho-ENO1 [37]. This supports the concept that ENO1-directed immunity is influenced, at least in part, by HLA genotype rather than arising solely from stochastic immune activation. From a translational perspective, these observations are highly significant. They imply that patient HLA typing could help predict or stratify immune responses against ENO1 and that phosphorylated ENO1 peptides could be considered in the design of vaccines or immune-monitoring strategies [37].

Together, these observations indicate that post-translational modifications such as citrullination and phosphorylation expand the antigenic landscape of ENO1 and contribute to inter-individual variability in immune recognition, supporting the view of ENO1 as a dynamic source of PTM-dependent neo-epitopes.

Among the post-translational modifications discussed in this review, citrullination and phosphorylation directly expand the antigenic repertoire of ENO1 by generating neo-epitopes recognized by adaptive immune cells. Other PTMs, although not directly immunogenic, profoundly influence ENO1 localization, stability, molecular interactions, and checkpoint regulation, thereby indirectly shaping tumor immunity. For clarity, the major ENO1 post-translational modifications discussed throughout the different sections of this review, together with their principal biological and immunological implications, are summarized in Table 1.

## 4. How ENO1 Shapes Innate Immune Programs and Antigen Accessibility in the Tumor Microenvironment

**Figure 3:**
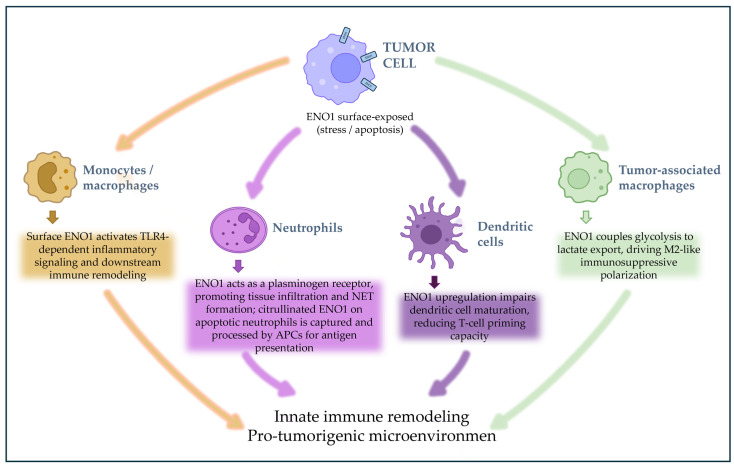
ENO1-dependent remodeling of innate immune programs within the tumor microenvironment. Immune-accessible ENO1 exposed during cellular stress, apoptosis, or inflammatory remodeling can modulate multiple innate immune cell populations through distinct but convergent mechanisms. In monocytes and macrophages, surface ENO1 activates TLR4-dependent inflammatory signaling programs associated with innate immune remodeling. In neutrophils, ENO1 functions as a plasminogen receptor that promotes tissue infiltration and NET formation, while ENO1 exposed on apoptotic neutrophils can become immunologically accessible during cell turnover. In dendritic cells, ENO1 upregulation is associated with impaired maturation and reduced T-cell priming capacity. In tumor-associated macrophages, ENO1-dependent glycolytic and lactate-associated pathways promote M2-like immunosuppressive polarization. Collectively, these interconnected mechanisms converge toward remodeling of the tumor inflammatory microenvironment under tumor-associated conditions. Abbreviations: APC, antigen-presenting cell; NET, neutrophil extracellular trap; TAM, tumor-associated macrophage; TLR4, Toll-like receptor 4.

Building on the determinants of ENO1 immunogenicity discussed above, this section examines how immune-accessible ENO1 can functionally modulate immune responses within the tumor microenvironment (Figure 3). In addition to serving as a source of antigenic epitopes, ENO1 can shape immune responses when exposed during cellular stress, tissue remodeling, or cell death.

### 4.1. ENO1 as a Putative DAMP-like Signal

When intracellular proteins become exposed or released during cellular stress or damage, they can function as damage-associated molecular pattern (DAMP)-like molecules that act as endogenous signals activating innate immune responses upon tissue injury. ENO1 has been shown to activate human monocytes through a CD14-dependent TLR4 signaling pathway, inducing an early wave of pro-inflammatory cytokines followed by delayed production of regulatory mediators such as IL-10 and IL-1 receptor antagonist. This biphasic response supports the possibility that extracellular ENO1 may function as an endogenous activator of innate immune receptors, shaping both inflammatory initiation and subsequent immune modulation [48].

Additional in vitro studies in human monocytes and macrophages have shown that engagement of cell-surface ENO1 with specific antibodies or plasminogen can trigger pro-inflammatory signaling cascades, including p38 MAPK and NF-κB activation, and promote the release of cytokines such as TNF-α, IL-1β and IL-6 [49,50]. Together with the demonstration that soluble ENO1 activates CD14-dependent TLR4 signaling in human monocytes [48], these complementary studies indicate that extracellular ENO1 can engage innate immune pathways through multiple non-canonical mechanisms depending on its mode of presentation.

Whether ENO1 operates as a DAMP-like signal in vivo within tumor microenvironments remains to be fully defined. Nevertheless, recent studies in acute inflammatory models further support this possibility. Lu et al. reported increased circulating ENO1 levels in inflammatory models and showed that ENO1 blockade attenuated inflammatory responses in vivo, consistent with previous studies indicating that extracellular ENO1 can activate monocytes/macrophages through CD14–TLR4 signaling [51]. Current evidence derives from complementary experimental approaches, including mechanistic studies in human monocytes and macrophages and acute inflammatory models in vivo; however, direct evidence demonstrating this mechanism within tumor microenvironments is still lacking. Together, these observations support the concept that extracellular ENO1 actively shapes myeloid inflammatory responses through innate immune receptor signaling, thereby contributing to inflammatory remodeling of the tumor microenvironment.

### 4.2. ENO1 Exposure During Neutrophil Turnover and Innate Immune Remodeling

Neutrophils represent a prominent and dynamic population in many tumor microenvironments. Their continuous recruitment, activation, and turnover contribute substantially to tissue remodeling and inflammatory flux within tumors [52,53]. In this setting, neutrophil apoptosis and clearance are integral components of ongoing inflammatory dynamics.

Alongside their role in inflammatory turnover, neutrophils also actively exploit surface ENO1 to support their own recruitment: inflammatory stimulation induces rapid redistribution of ENO1 to the neutrophil surface, where it functions as a plasminogen receptor that facilitates pericellular proteolysis and tissue transmigration. Antibody-mediated blockade of surface ENO1 significantly reduces neutrophil infiltration in vivo and limits the formation of neutrophil extracellular traps (NETs), web-like DNA–protein structures released by activated neutrophils. Consistent with these effects, ENO1 inhibition also reduces inflammatory tissue damage, supporting a direct role for ENO1 in neutrophil trafficking and inflammatory amplification [51].

In the tumor microenvironment, neutrophil turnover may provide a biologically relevant setting in which these mechanisms operate.

ENO1, including citrullinated ENO1 species, is exposed on the surface of apoptotic neutrophils, rendering this otherwise intracellular protein directly accessible to immune capture [17]. Because apoptotic neutrophils are efficiently cleared by macrophages and dendritic cells through efferocytosis, the physiological removal of dying cells by phagocytes, ENO1-derived material can potentially be transferred to professional antigen-presenting cells and become available for intracellular antigen processing and presentation [17].

Through this mechanism, neutrophil turnover can influence the local inflammatory state of the tumor microenvironment, shaping the quality and intensity of innate immune signaling and indirectly affecting downstream immune responses. While neutrophils can exert both protumor and antitumor functions depending on context, whether ENO1 exposure during neutrophil turnover plays a causal role in directing these outcomes remains an open and conceptually relevant question that requires further investigation [17,52,53].

Thus, current evidence links ENO1 to neutrophil biology through distinct but not yet fully integrated lines of investigation: functional studies in acute inflammation define a role for surface ENO1 in neutrophil recruitment [51], whereas apoptotic-neutrophil studies mainly support ENO1 exposure as a potential source of immune-accessible antigen [17].

In addition to its role in antigen exposure, neutrophils can also influence ENO1 expression in tumor cells through paracrine signaling mechanisms. A distinct subset of tumor-infiltrating C5aR1^+^ neutrophils, characterized by expression of the complement receptor C5aR1 and associated with inflammatory and tumor-promoting functions, has been shown to increase ENO1 levels in breast cancer cells. This effect is mediated by the release of inflammatory cytokines such as IL-1β and TNFα, which activate ERK1/2-dependent pathways and stabilize *ENO1* transcripts via m6A RNA modification, ultimately reinforcing glycolytic metabolism [54].

Clinically, the co-occurrence of high ENO1 expression and signatures of C5aR1^+^ neutrophil infiltration has been associated with poorer patient outcomes, supporting the idea that neutrophil–tumor crosstalk can influence the metabolic and immunological landscape of the tumor microenvironment in an ENO1-dependent manner [54].

### 4.3. ENO1 and Dendritic Cell Function

Dendritic cell activation is tightly linked to metabolic reprogramming, including enhanced glycolytic activity. In multiple myeloma, interactions between tumor cells and plasmacytoid dendritic cells (pDCs) were shown to increase ENO1 expression in both cell populations, and elevated ENO1 levels were associated with impaired immune stimulatory activity of pDCs [55]. Pharmacologic inhibition of ENO1 increased the expression of pDC activation/maturation markers, including CD80, CD83, and CD40, and enhanced pDC-mediated activation of cytotoxic T and NK cells. These findings support an immunomodulatory role for ENO1 in the tumor microenvironment and suggest that ENO1 upregulation contributes to a less immunogenic dendritic cell state.

Although current evidence remains limited, these observations suggest that ENO1-dependent metabolic programs may influence dendritic cell functionality and antigen-presenting capacity within tumor-associated immune environments.

At present, however, these conclusions are largely derived from multiple myeloma models, and whether comparable ENO1-dependent mechanisms regulate dendritic-cell function across other tumor types remains to be established.

### 4.4. ENO1 and Macrophage Recruitment and Polarization

Macrophages are central regulators of tumor-associated inflammation and immune suppression, and their functional polarization critically influences tumor progression. Emerging evidence indicates that ENO1 participates in molecular pathways that shape macrophage behavior through both tumor-intrinsic and immune cell-intrinsic mechanisms.

In tumor cells, a circular RNA derived from the *FUT8* locus (*circFUT8*) directly binds ENO1, forming a cytoplasmic RNA–protein complex that enhances ENO1-dependent glycolysis. This interaction increases the production of glycolytic metabolites, particularly lactate and ATP. Elevated lactate levels act as metabolic signals that promote activation of TNF-related pathways and the expression of macrophage-modulating factors such as CSF1 and CCL5. These tumor-derived metabolic and cytokine cues collectively promote macrophage polarization toward an M2-like protumor phenotype, thereby linking ENO1 to metabolic regulation of the tumor immune microenvironment [56].

While these mechanisms have been primarily characterized in tumor settings, evidence from non-oncologic inflammatory models offers a broader perspective on ENO1-dependent macrophage programming. Within macrophages, mechanistic studies from inflammatory models indicate that ENO1 contributes to the metabolic reprogramming underlying pro-inflammatory activation. Under inflammatory conditions, HIF-1α upregulates glycolytic enzymes including ENO1, thereby sustaining the glycolytic flux required for M1-associated cytokine production. Genetic or pharmacological inhibition of ENO1 dampens glycolysis and reduces inflammatory cytokine release, identifying ENO1 as a metabolic effector that supports macrophage inflammatory programming [57]. These distinct effects likely reflect context-dependent roles of ENO1 in myeloid cells, where metabolic programming can support either pro-inflammatory or immunosuppressive phenotypes depending on environmental cues, and may in part relate to differences between tumor-associated and inflammatory settings.

Consistent with a broader role for ENO1 in macrophage metabolic adaptation, proteomic analyses of tumor-associated macrophages (TAMs) show that ENO1 is upregulated as part of a glycolytic program during their differentiation, together with key enzymes such as hexokinase-2 and phosphofructokinase. These data indicate that ENO1 upregulation is a reproducible feature of metabolically reprogrammed TAMs and not limited to isolated inflammatory settings [58].

Further in vivo evidence indicates that ENO1 can also influence the recruitment of inflammatory myeloid populations. In prostate cancer models, antibody-mediated targeting of surface ENO1 reduced the infiltration of CCR2^+^ inflammatory monocytes, which can differentiate into tumor-associated macrophages, without markedly altering total macrophage numbers. This finding suggests a selective effect on pro-tumorigenic myeloid subsets. This targeting was also accompanied by decreased production of chemokines such as CCL2 and immunomodulatory factors including TGF-β, supporting a role for ENO1 in shaping cytokine networks that regulate myeloid cell trafficking within the tumor microenvironment [59].

Supporting a direct role for surface ENO1 in regulating myeloid cell trafficking, studies in non-oncologic inflammatory settings have shown that antibody-mediated blockade of surface ENO1 reduces the recruitment of monocytes, macrophages, dendritic cells, and neutrophils, together with decreased levels of chemokines such as CCL2 and IL-8. These effects were linked to impaired ENO1-dependent plasminogen activation and pericellular proteolysis, highlighting a mechanism by which surface ENO1 can facilitate immune cell migration [60].

In glioblastoma models, ENO1-high tumor cells were shown to promote the establishment of an immunosuppressive microglial/macrophage program. Functional co-culture experiments demonstrated that ENO1 expression in tumor cells enhanced markers associated with tumor-supportive polarization, linking tumor glycolytic activity to local immune regulation [61].

Additional mechanistic insight has recently emerged from studies linking ENO1 surface localization to lactate-dependent myeloid programming. Membrane-associated ENO1 was shown to interact functionally with the lactate transporter MCT4, facilitating efficient coupling between glycolytic flux and lactate export by tumor cells. Elevated extracellular lactate concentrations promoted macrophage polarization toward an M2-like phenotype, reinforcing an immunosuppressive microenvironment. Interference with ENO1 surface localization or antibody-mediated targeting of surface ENO1 reduced lactate release and shifted macrophage polarization toward a more pro-inflammatory profile. These findings indicate that surface-exposed ENO1 functionally connects tumor glycolysis with the regulation of myeloid cell responses within the tumor microenvironment [35]. Although these findings identify surface ENO1 as a promising therapeutic target, their translational relevance remains to be validated in additional tumor models and, ultimately, in clinical studies.

Together, these findings identify ENO1 as a key regulator linking tumor glycolysis, myeloid-cell programming, and microenvironmental remodeling, providing the basis for the immune suppressive mechanisms discussed in the following section.

## 5. How ENO1 Drives Immune Suppression and Immune Escape

**Figure 4:**
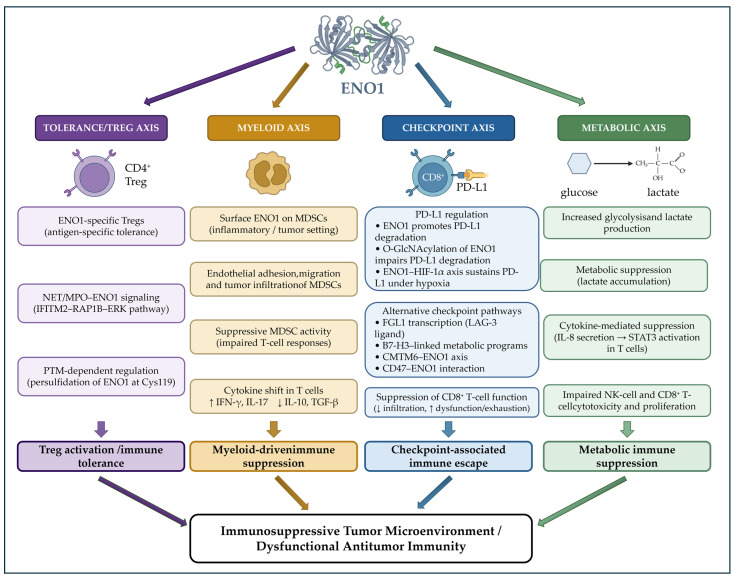
Integrated model of ENO1-mediated mechanisms of tumor immune escape. ENO1 contributes to tumor immune escape through four interconnected mechanisms. The tolerance axis includes ENO1-dependent pathways that promote immune tolerance and regulatory T-cell responses. The myeloid axis involves the recruitment and immunosuppressive activity of myeloid cell populations. The checkpoint axis summarizes the interaction between ENO1 and multiple immune checkpoint pathways that limit antitumor immunity. The metabolic axis reflects ENO1-driven glycolytic reprogramming and lactate accumulation, leading to impaired effector immune-cell function. Together, these interconnected mechanisms converge to establish an immunosuppressive tumor microenvironment and dysfunctional antitumor immunity. Abbreviations: PD-L1, programmed death-ligand 1; Treg, regulatory T cell.

ENO1-dependent myeloid reprogramming converges with additional mechanisms that impair adaptive immune control, including antigen-specific tolerance, suppressive immune circuits, and tumor-intrinsic pathways intersecting with immune checkpoint regulation. Rather than acting as a universal driver of immune escape, ENO1 functions as a context-dependent immunological modulator integrated within metabolic, inflammatory, and checkpoint-related pathways. These interconnected mechanisms are schematically summarized in Figure 4.

### 5.1. ENO1-Directed Antigen-Specific Tolerance and Treg Programming

ENO1 may contribute to tumor immune escape by promoting antigen-specific tolerance. ENO1-overexpressing tumors have been shown to induce regulatory T cells that selectively suppress anti-ENO1 immune responses, indicating that tolerance to this antigen can arise in a highly targeted manner [28]. Notably, ex vivo analyses of tumor-infiltrating lymphocytes in PDAC have shown enrichment of ENO1-specific Treg clones within tumor tissue compared with healthy pancreatic tissue, where ENO1-specific responses display a more Th1-oriented profile. This compartmentalized distribution suggests that the tumor microenvironment biases ENO1-specific immunity toward regulatory T-cell responses rather than effector T-cell activity [62]. In line with this concept, regulatory T cells specific for ENO1 capable of suppressing effector responses directed against the same antigen have been described in cancer patients, indicating that tolerance to ENO1 can arise in an antigen-specific manner and may contribute to tumor immune escape [24].

In addition to antigen-specific tolerance mechanisms, ENO1 can also influence Treg differentiation through antigen-independent signaling pathways. Mechanistic evidence indicates that ENO1 can promote regulatory T-cell differentiation through cell-surface signaling mechanisms. Surface-exposed ENO1 on CD4^+^ T cells serves as a receptor for myeloperoxidase (MPO) associated with NETs, which are commonly generated in inflammatory microenvironments. Binding of NET-associated MPO to ENO1 promotes recruitment of the transmembrane protein interferon-induced transmembrane protein 2 (IFITM2), which binds NET-derived DNA and transduces signals to the RAP1B–ERK pathway, thereby promoting Treg differentiation and functional activation. Although described in acute inflammatory settings, this pathway indicates that ENO1 can actively participate in Treg programming. Given the presence of NETs and MPO-rich inflammatory microenvironments in both inflammatory and tumor contexts, this mechanism suggests a plausible route by which NET–T-cell interactions may favor regulatory immune states [63].

Building on the PTM-dependent mechanisms outlined in Section 3, recent evidence from colorectal cancer further expands the spectrum of ENO1 post-translational regulation in immune contexts, indicating that hydrogen sulfide–induced persulfidation of ENO1 at Cys119 can promote regulatory T-cell activation and contribute to an immunosuppressive tumor microenvironment in colorectal cancer [64] (Table 1).

Together, evidence from patient specimens, experimental tumor models, and mechanistic studies indicates that ENO1 can promote regulatory immune states through antigen-restricted Treg responses, ENO1-dependent surface signaling pathways, and post-translationally regulated mechanisms, creating conditions that favor tumor immune escape.

### 5.2. Myeloid Axis: ENO1-Dependent Programming of Suppressive Myeloid Compartments

ENO1 can also contribute to tumor immune escape through its effects on myeloid-derived suppressor cells (MDSCs), which represent a major barrier to productive antitumor immunity in multiple cancer settings. ENO1 has been reported to be aberrantly expressed at the surface of MDSCs, especially under inflammatory and tumor-associated conditions, where it supports endothelial adhesion, migration, and tumor infiltration. Notably, antibody-mediated targeting of ENO1 impairs MDSC trafficking, attenuates suppressive activity, and restores effector T-cell function, identifying surface-exposed ENO1 as a functional determinant of MDSC-mediated immune restraint in vivo [65,66].

Mechanistically, antibody-mediated targeting of surface ENO1 on MDSCs not only limits their trafficking but can also shift downstream T-cell responses toward a more effector-oriented profile. T cells cultured in the presence of MDSCs treated with anti-ENO1 antibodies produce higher IFN-γ and IL-17 and lower IL-10 and TGF-β [62]. This cytokine pattern is consistent with the possibility that ENO1-expressing MDSCs skew antitumor T-cell responses toward regulatory rather than effector functions, in agreement with the established suppressive activity of MDSCs [62]. Together, these observations support a functional link between ENO1 immune accessibility on suppressive myeloid cells and inhibition of antitumor T-cell responses.

### 5.3. Checkpoint Axis: ENO1 Integration with Immune Checkpoint Regulation

ENO1 intersects with immune checkpoint regulation through at least four partially overlapping axes: direct and PTM-dependent modulation of PD-L1 stability and surface expression; transcriptional regulation of alternative checkpoint ligands, including FGL1 and B7-H3; functional association with membrane-stabilizing complexes such as CKLF-like MARVEL transmembrane domain-containing protein 6 (CMTM6); and integration within the CD47 signaling network. These mechanisms operate across distinct tumor contexts and are discussed in turn below (Table 2).

#### 5.3.1. PD-L1-Centered Regulation

Available evidence indicates that ENO1 regulates PD-L1 through multiple context-dependent mechanisms operating at distinct regulatory levels across different tumor settings.

Transcriptomic analyses in cervical cancer initially identified positive correlations between ENO1 expression and immunosuppressive molecules including PD-L1 and TGF-β1, supporting the association of ENO1-high tumors with suppressive immune states [67].

**Table 2:** ENO1-associated immune checkpoint axes in cancer.

Checkpoint Axis	Mechanism	Cancer Model/Tumor Context	[Ref.]
PD-L1 (direct)	ENO1 promotes PD-L1 ubiquitination/degradation	NSCLC, lung cancer	[68]
PD-L1 (PTM-dependent)	O-GlcNAcylation of ENO1 impairs STUB1 recruitment, stabilizes PD-L1	Colorectal cancer	[69]
PD-L1 (indirect, HIF-1α)	ENO1 sustains HIF-1α, which maintains PD-L1 expression	PDAC	[70]
Anti-PD-L1 resistance (SPP1-TAM axis)	ENO1 drives SPP1-mediated immunosuppression and anti-PD-L1 resistance	Bladder cancer	[71]
FGL1/LAG-3	Nuclear ENO1 drives FGL1 transcription	Intrahepatic cholangiocarcinoma	[72]
B7-H3	ENO1 supports B7-H3-linked glycolytic programs	Lung cancer	[73]
CMTM6	CMTM6 stabilizes membrane-associated ENO1 and activates the ENO1–AKT/GSK3β–Wnt signaling axis	Cisplatin-resistant OSCC	[74]
CD47	CD47 stabilizes ENO1 via FBXW7 suppression; links phagocytosis checkpoint to glycolysis	Colorectal cancer	[75]

Mechanistic studies subsequently demonstrated that ENO1 can directly influence PD-L1 stability. In immunogenic tumor models including melanoma and lung carcinoma, ENO1 has been shown to interact with PD-L1 and promote its ubiquitination and proteasomal degradation, thereby limiting PD-L1 stability and surface expression on tumor cells. The immunological relevance of this pathway is supported by the observation that ENO1-dependent modulation of PD-L1 influences tumor control in immunocompetent but not immunodeficient models, indicating a direct impact on adaptive immune responses in vivo [68].

More recent evidence indicates that the impact of ENO1 on PD-L1 is strongly influenced by post-translational modification, consistent with PTM-dependent regulatory mechanisms. In tumors characterized by high glucose metabolism, including hepatocellular carcinoma, O-GlcNAcylation of ENO1 at specific serine residues has been shown to weaken the interaction between ENO1 and PD-L1 and to impair recruitment of the E3 ligase STUB1, thereby reducing PD-L1 ubiquitination and prolonging its stability on tumor cells. In this modified state, ENO1 indirectly supports sustained PD-L1 surface expression and tumor immune evasion [69] (Table 1).

In PDAC, ENO1 has also been reported to promote immune escape by indirectly sustaining PD-L1 expression in hypoxic tumors. In orthotopic PDAC models, *ENO1* knockdown reduced HIF-1α levels and was accompanied by lower PD-L1 expression, increased intratumoral CD8^+^ T-cell infiltration, and enhanced CD8^+^ effector readouts (including granzyme B and IFN-γ/TNF-α production). Pharmacologic stabilization of HIF-1α rescued PD-L1 expression in *ENO1*-silenced cells, supporting an ENO1–HIF-1α axis that can maintain PD-L1–mediated T-cell dysfunction in this setting [70].

Rather than representing mutually exclusive mechanisms, the available studies likely describe different regulatory layers operating in distinct biological contexts. The positive association observed in transcriptomic datasets does not establish a direct causal relationship between ENO1 and PD-L1 expression, but more likely reflects the broader immunosuppressive phenotype of ENO1-high tumors. By contrast, mechanistic studies indicate that unmodified or functionally competent ENO1 can directly interact with PD-L1 and promote its STUB1-dependent ubiquitination and proteasomal degradation. In this setting, ENO1 acts as a negative regulator of PD-L1 stability. This mechanism can be altered by the post-translational state of ENO1: O-GlcNAcylation weakens the ENO1–PD-L1 interaction, reduces STUB1-dependent PD-L1 ubiquitination and degradation, and thereby favors PD-L1 stabilization. A further level of regulation is suggested by PDAC models, in which ENO1 indirectly sustains PD-L1 expression through the HIF-1α pathway. Collectively, these observations suggest that the net effect of ENO1 on PD-L1 depends not only on ENO1 abundance, but also on tumor metabolic state, hypoxic signaling, post-translational modification status, and the regulatory level being examined. In this framework, ENO1 can either facilitate PD-L1 degradation or support PD-L1 persistence, depending on the molecular context.

#### 5.3.2. Checkpoint-Associated Suppressive Circuits Beyond PD-L1

ENO1-dependent checkpoint regulation extends beyond direct PD-L1 control. A CRISPR-based in vivo screen in bladder cancer models identified ENO1 as a determinant of resistance to anti-PD-L1 therapy. *ENO1*-deficient tumors displayed increased CD8^+^ T-cell infiltration and effector function, together with reduced tumor growth under checkpoint blockade [71]. Mechanistically, ENO1 was shown to enhance the expression of secreted phosphoprotein 1 (SPP1, also known as osteopontin) by stabilizing its mRNA through direct binding to the *SPP1* 3′UTR. Elevated SPP1 promoted polarization of tumor-associated macrophages toward an M2-like phenotype and directly impaired CD8^+^ T-cell function via ITGA4/ITGB1 signaling. This created a feed-forward immunosuppressive circuit in which both tumor cells and M2 macrophages contributed to SPP1 production, collectively limiting cytotoxic T-cell activity in the tumor microenvironment [71]. Notably, genetic or pharmacologic ENO1 inhibition synergized with anti-PD-L1 therapy, resulting in stronger CD8^+^ T-cell responses and improved tumor control. These findings identify ENO1 as an upstream metabolic and post-transcriptional regulator of checkpoint responsiveness. Its effects appear to involve remodeling of cytokine and myeloid programs rather than regulation of checkpoint expression alone [71].

Importantly, many of these mechanisms have been characterized in defined experimental models, and their relative contribution in human cancers likely varies across tumor types and clinical contexts.

Emerging evidence suggests that ENO1 may intersect with multiple non–PD-L1 checkpoint pathways. In intrahepatic cholangiocarcinoma models, ENO1 was shown to translocate to the nucleus and bind the promoter of *FGL1*, a ligand for LAG-3, an inhibitory receptor expressed on activated T cells, thereby promoting *FGL1* transcription and reducing CD8^+^ T-cell effector function. These findings further support a role for ENO1 as a transcriptional regulator linking tumor metabolic programs to immune checkpoint control [72].

In this broader checkpoint context, ENO1 has been reported to functionally associate with B7-H3 (CD276), a member of the B7 family frequently overexpressed in tumors and known to dampen T-cell responses. In this context, ENO1 appears to support B7-H3–linked glycolytic programs in cancer cells, pointing to a connection between checkpoint signaling and metabolic regulation that may indirectly influence antitumor immunity [73].

In parallel, ENO1 has been functionally linked to the CMTM6 axis. In cisplatin-resistant carcinoma models, CMTM6 was shown to physically associate and co-localize with plasma membrane-associated ENO1, and *CMTM6* silencing reduced ENO1 abundance in the membrane fraction, consistent with a role for CMTM6 in stabilizing surface-exposed ENO1. Functionally, this CMTM6–ENO1 connection was coupled to activation of the AKT/GSK3β/β-catenin pathway and Wnt signaling outputs associated with chemoresistance [74]. Given that CMTM6 is a recognized regulator of PD-L1 stability and membrane trafficking, these findings raise the possibility that ENO1-containing CMTM6 complexes may intersect with checkpoint-relevant trafficking programs, although this link has not been directly tested in the ENO1–CMTM6 setting.

Additional layers of immune regulation emerge from ENO1 integration within the CD47 axis. CD47 is a transmembrane “don’t eat me” signal that restrains macrophage phagocytosis via SIRPα, but it can also exert tumor-intrinsic functions. In colorectal cancer models, CD47 was shown to physically interact with ENO1 and protect ENO1 from proteasomal turnover by suppressing FBXW7-dependent ENO1 ubiquitination, thereby stabilizing ENO1 protein levels. Functionally, this CD47–ENO1 coupling increased glycolytic outputs and promoted ERK phosphorylation in cancer cells [75]. In this setting, the CD47–ENO1 interaction links a canonical phagocytosis checkpoint molecule to ENO1-dependent metabolic and pro-migratory signaling programs within tumor cells, providing a concrete molecular connection between checkpoint-associated receptors and metabolic adaptation [75] (Table 1).

### 5.4. Metabolic Axis: ENO1-Driven Immunometabolic Suppression

Recent work has identified an additional ENO1-dependent pathway of tumor-driven T-cell suppression. In oral squamous cell carcinoma, ENO1 was shown to interact with apolipoprotein C-III (ApoC3) and to promote tumor-cell secretion of IL-8. Tumor-derived IL-8 activated STAT3 signaling in T cells, resulting in reduced proliferation and increased apoptosis, thereby establishing a cytokine-mediated mechanism of ENO1-driven immune escape [76].

An additional layer of ENO1-driven immune regulation has recently been linked to metabolic interactions within the tumor stroma. In triple-negative breast cancer models, syndecan-1 (SDC1) expressed by cancer-associated fibroblasts was shown to bind the TIM-barrel catalytic domain of ENO1 and prevent its FBXW7-mediated ubiquitination and proteasomal degradation. This interaction stabilized ENO1, increased glycolytic flux, and promoted lactate accumulation in the tumor microenvironment. The resulting lactate-rich milieu impaired the cytotoxic activity of both NK cells and CD8^+^ T cells, whereas pharmacologic inhibition of ENO1 or blockade of lactate export restored immune cytotoxicity and radiosensitivity. These findings identify a stromal ENO1-dependent metabolic circuit capable of simultaneously promoting tumor stemness, immune suppression, and therapy resistance [77] (Table 1). A conceptually related mechanism has recently been described in HER2-positive breast cancer, where the inflammatory protein S100A9 was shown to directly interact with and stabilize ENO1, thereby enhancing glycolytic activity and lactate production. Increased ENO1-dependent metabolic flux was associated with the establishment of an immunosuppressive microenvironment and promoted tumor progression, further supporting the view that ENO1 functions as a central integrator of inflammatory and metabolic signals that converge on immune regulation within the tumor microenvironment [78].

More broadly, ENO1 operates within a metabolic landscape that is itself a major determinant of antitumor immunity. Metabolic constraints characteristic of the tumor microenvironment, including nutrient competition, hypoxia, and extracellular acidification, are well-recognized drivers of immune dysfunction and immune suppression [22]. For instance, competition for glucose between highly glycolytic tumor cells and infiltrating T lymphocytes can limit T-cell proliferation and cytokine production, whereas hypoxic and acidic conditions can impair antigen-presenting cell function and favor T-cell exhaustion [22]. Consistent with this concept, impaired glycolytic fitness in tumor-infiltrating CD8^+^ T cells has been associated with defective effector function, highlighting the importance of sustained metabolic competence for effective antitumor immunity [79]. As a central glycolytic enzyme frequently upregulated in tumors, ENO1 may contribute to maintaining the metabolic constraints imposed by highly glycolytic tumor microenvironments and thereby indirectly limit the effectiveness of adaptive immune responses, including cytotoxic T-cell activity [22,80].

Consistent with this broader immunometabolic framework, recent single-cell and spatial multi-omics analyses in prostate cancer have linked ENO1-driven glycolytic programs to immunosuppressive tumor microenvironments characterized by enrichment of regulatory T cells and macrophage-associated stromal remodeling, with higher ENO1 expression further correlating with lymph node metastatic disease [81]. These clinical and multi-omics observations reinforce the concept that ENO1-dependent metabolic reprogramming is not confined to experimental models but associates with immunosuppressive microenvironmental states in human tumors. However, the immunological consequences of ENO1 expression may not be uniformly suppressive across all tumor contexts. A large breast cancer cohort study reported that high ENO1 expression was associated with increased infiltration of CD8^+^ T cells, NK cells, B cells, and M1 macrophages and correlated with improved survival in stage I–II disease, suggesting that the impact of ENO1 on tumor immunity may depend on tumor type, disease stage, and microenvironmental context [82].

The immunometabolic reach of ENO1 may extend beyond immune cell populations to encompass stromal compartments of the tumor microenvironment. In multiple myeloma models, ENO1-dependent metabolic reprogramming—mediated in part through plasmin activation and TGF-β signaling—was shown to drive cancer-associated fibroblast differentiation, altering the metabolic and cytokine landscape of the tumor stroma in ways that support disease progression and therapeutic resistance [83]. These observations suggest that ENO1-driven metabolic programs can propagate immunosuppressive and pro-tumorigenic signals beyond immune cell populations, contributing to a broader remodeling of the tumor microenvironment.

Collectively, current evidence positions ENO1 as a multifunctional contributor to immune regulation in cancer, whose impact depends on tumor context, metabolic state, and microenvironmental cues. ENO1 can act within tumor cells by regulating metabolic adaptation and immune checkpoint availability [68,75], and within the immune compartment by shaping macrophage programming and MDSC function [56,57,65]. Rather than driving immune escape through a single dominant pathway, ENO1 operates as a context-dependent integrator of metabolic reprogramming, myeloid polarization, and checkpoint regulation, a functional convergence that, under tumor-associated conditions, consistently tilts toward immune suppression (Figure 4).

## 6. Therapeutic Targeting of ENO1: Preclinical Evidence

The mechanisms described in the preceding section—encompassing MDSC-mediated immune restraint, ENO1-driven checkpoint regulation, metabolic suppression of cytotoxic lymphocytes, and antigen-specific Treg programming—collectively define a set of functionally accessible nodes for therapeutic intervention. The strategies reviewed below are organized around the modalities through which ENO1 has been targeted preclinically: antibody-based blockade of surface-exposed ENO1, active immunization through ENO1-directed vaccines, and pharmacologic inhibition of ENO1 enzymatic activity. Where relevant, the mechanistic rationale for each approach is explicitly connected to the immunosuppressive circuits described above.

### 6.1. Antibody-Based Strategies

Antibody-based strategies targeting ENO1 have provided proof-of-concept evidence that surface-exposed ENO1 represents a functionally relevant and therapeutically accessible pool. Early work showed that targeting cell-surface ENO1 can interfere with plasminogen binding and the downstream plasmin-dependent proteolytic cascade, thereby limiting pericellular matrix remodeling and invasion-related migratory behavior [9,11,84,85]. These findings support the concept that cell-surface ENO1 is not merely an epiphenomenon of transformation but can serve as a functional driver of proteolysis-linked tissue remodeling programs amenable to therapeutic blockade [9,11,85].

Beyond direct effects on tumor cells, ENO1-directed antibodies exert immunomodulatory activity through myeloid compartments. As detailed in Section 5.2, surface ENO1 on MDSCs supports their trafficking and suppressive function; antibody-mediated targeting of this pool limits MDSC accumulation in vivo and indirectly restores effector T-cell activity [65]. These findings position anti-ENO1 antibodies as dual-function agents capable of simultaneously disrupting ENO1-dependent pericellular remodeling and relieving myeloid-driven immune suppression.

In the context of combination strategies, recent preclinical studies in colorectal and triple-negative breast cancer models have shown that radiotherapy increases ENO1 surface exposure through TGF-β signaling, thereby potentiating the efficacy of antibody-based ENO1 targeting. Treatment with the anti-ENO1 antibody HuL001 in combination with radiotherapy significantly improved tumor control and increased rates of complete responses [35], providing proof-of-concept for ENO1 as a combinatorial immunometabolic target.

Collectively, these studies provide proof-of-concept that ENO1-directed antibodies can simultaneously target tumor-intrinsic and immune-suppressive mechanisms [11,65]. In addition, sustained in vivo delivery of anti-ENO1 antibodies through adeno-associated viral vector (AAV)-mediated antibody gene transfer has been shown to produce long-lasting circulating antibody levels and to reduce metastatic burden more effectively than repeated antibody injections, supporting the feasibility of prolonged ENO1-targeted passive immunotherapy [66].

### 6.2. ENO1-Based Vaccines

#### 6.2.1. ENO1 Vaccination and Immune Remodeling

Vaccination strategies targeting ENO1 represent the most advanced and mechanistically developed preclinical application of ENO1-directed immunotherapy. An early preclinical study employed a DNA-based vaccine encoding full-length ENO1 and demonstrated that ENO1 vaccination induces coordinated humoral and cellular immune responses in genetically engineered mouse models of pancreatic ductal adenocarcinoma (PDAC) [86]. Vaccinated animals exhibited prolonged survival, reduced tumor progression, decreased metastatic dissemination, and evidence of epitope spreading toward additional tumor-associated antigens, providing direct evidence that ENO1-directed immune priming can be translated into measurable antitumor activity in vivo.

From a mechanistic standpoint, ENO1 DNA vaccination elicited robust anti-ENO1 antibody responses together with antigen-specific CD4^+^ T-cell immunity characterized by Th1/Th17-skewed responses. This coordinated humoral and cellular immune architecture supports antigen-specific helper T-cell activation rather than isolated serological reactivity and is consistent with a model in which ENO1 vaccination promotes integrated adaptive immune engagement [86,87]. Notably, anti-ENO1 antibodies induced by vaccination have also been shown to directly kill ENO1-expressing tumor cells through complement-dependent cytotoxicity, indicating that humoral responses can contribute not only to immune recognition but also to direct tumor cell elimination [66].

Importantly, clinical observations indicate that ENO1-directed immunity can also arise dynamically in patients during conventional cancer treatment. In PDAC patients receiving chemotherapy, repeated analyses of peripheral blood samples revealed treatment-associated changes in ENO1-specific T-cell responses over time. Chemotherapy promoted both the expansion of pre-existing ENO1-reactive T-cell clones and the emergence of new ENO1-specific clonotypes, indicating ongoing antigen-driven immune selection. Importantly, ENO1-specific T cells detected after treatment were functionally active and capable of producing pro-inflammatory cytokines such as IFN-γ, consistent with antitumor effector functions. These findings suggest that chemotherapy can create conditions that favor the priming and amplification of ENO1-directed immunity, likely through increased antigen availability and/or improved immune priming. Beyond confirming the clinical relevance of ENO1 as a naturally targeted antigen, these observations provide a translational rationale for integrating ENO1-directed immunotherapies with treatments that enhance antigen exposure and immune priming [88].

Consistent with these observations, vaccination was associated with remodeling of the tumor immune microenvironment, including reduced accumulation of immunosuppressive populations such as MDSCs and regulatory T cells, thereby shifting local immune balance toward a state more permissive for immune-mediated tumor control [86]. Notably, in pancreatic cancer models, ENO1 vaccination was also shown to induce the formation of tertiary lymphoid structures (TLS) within tumors, organized lymph node–like aggregates where B cells, T cells, and antigen-presenting cells cluster together, suggesting that vaccination can support local sites of immune activation directly inside the tumor [89].

Importantly, detailed analyses in pancreatic cancer models indicate that ENO1 vaccine-induced TLS are not merely architectural structures but functionally active immune niches. Within these TLS, B cells contribute to local antigen presentation and support CD8^+^ T-cell infiltration.

In human PDAC cohorts, B-cell-rich TLS correlated with improved prognosis and increased CD8^+^ T-cell density, highlighting a link between ENO1-driven humoral responses and cytotoxic immunity [62]. These observations suggest that ENO1 vaccination can favor the formation of structured, pro-immunogenic B-cell niches rather than merely increasing total B-cell infiltration.

These findings further indicate that ENO1 vaccination does not act solely by expanding antigen-specific lymphocyte pools but also indirectly reshapes suppressive myeloid circuits that otherwise constrain effector function in PDAC. This dual impact—on adaptive immune activation and on the immunosuppressive architecture of the tumor microenvironment—provides a mechanistic basis for the durable antitumor effects observed in vaccinated animals [86]. At the same time, experimental B-cell depletion studies indicate that not all tumor-infiltrating B cells are functionally equivalent, as dispersed B-cell infiltrates may exert context-dependent effects distinct from organized TLS [62].

#### 6.2.2. Optimization and Combination Strategies

Building on the concept of ENO1-targeted vaccination, more recent preclinical work has begun to explore refined vaccine designs aimed at focusing immune responses toward the most immunogenic ENO1 epitopes. A conference report describing a multi-epitope ENO1 DNA vaccine (ENO3PEP) in genetically engineered PDAC models indicated that epitope-focused vaccination can reproduce and potentially enhance several features observed with full-length ENO1 vaccines. ENO3PEP vaccination was associated with reduced primary tumor burden and metastatic dissemination, early induction of anti-ENO1 IgG responses with a Th1-associated IgG2c bias, and increased IFN-γ production by ENO1-reactive T cells. Notably, this approach was also reported to remodel the tumor microenvironment, with increased infiltration of CD4^+^ and CD8^+^ T cells and M1-like macrophages, reduced infiltration of FoxP3^+^ regulatory T cells, the canonical immunosuppressive CD4^+^ T-cell population, and M2-like macrophages, and decreased collagen deposition. These findings support the concept that rational epitope selection represents a viable strategy to further optimize ENO1-based vaccination platforms [90].

Subsequent studies demonstrated that ENO1-based vaccination can be potentiated through rational combination strategies designed to increase antigen availability or relieve dominant suppressive programs. Chemotherapy has been shown to enhance immune recognition of tumor-associated antigens and to synergize with ENO1 DNA vaccination [91]. These findings provide a rationale for combining ENO1 vaccination with standard cytotoxic regimens to amplify vaccine-induced immune responses and broaden antitumor immunity [91]. Notably, clinical and preclinical data indicate that chemotherapy does not simply increase antigen availability but can actively amplify pre-existing antitumor immunity. In patients with pancreatic cancer, chemotherapy was associated with higher levels and broader repertoires of antibodies against tumor-associated antigens, including ENO1. This finding indicates that standard treatments can reinforce ongoing immune recognition rather than merely induce nonspecific inflammation [91]. Mechanistically, this immune potentiation was accompanied by a shift toward effector T-cell responses and by evidence of antigen spreading, whereby immune responses initially directed against ENO1 extended to additional tumor antigens. In mouse models, administering gemcitabine prior to ENO1 vaccination significantly improved tumor control and revealed a critical contribution of CD4^+^ T cells to therapeutic efficacy [91]. This combination strategy is particularly relevant in PDAC, where baseline immune exclusion and suppressive myeloid infiltration frequently limit the efficacy of antigen-specific immune responses.

More recently, targeting suppressive myeloid signaling pathways has emerged as an additional strategy to enhance ENO1 vaccine efficacy. Inhibition of PI3Kγ in combination with ENO1 DNA vaccination was shown to potentiate antitumor immunity through mechanisms that included a prominent B-cell–dependent component, enhanced germinal center activity and antibody responses, as well as evidence of antigen spreading and improved intratumoral CD8^+^ T-cell infiltration and tumor control [92]. These data highlight that ENO1 vaccination can engage coordinated humoral and cellular immune programs when dominant innate suppressive checkpoints are relieved.

Importantly, at the level of antigen design, citrullinated ENO1 peptides (Section 3.1) offer a further refinement: the strong immunogenicity of post-translationally modified ENO1 epitopes, particularly citrullinated ENO1 peptides, provides a conceptual extension of these vaccination strategies. Consistent with the enhanced MHC class II binding and T-cell activation conferred by citrullination (Section 3.1 and Table 1), ENO1-directed vaccines incorporating modified peptides may achieve increased functional avidity and immune selectivity in tumor-associated contexts [16].

Building on the conceptual rationale for PTM-directed vaccination, recent work has provided direct experimental validation of this strategy. In a triple-negative breast cancer model, citrullinated ENO1 peptides were identified through immunopeptidomic and surface proteomic analyses and selected based on predicted MHC binding. Vaccination with these citrullinated ENO1 peptides elicited ENO1-specific T-cell responses, delayed tumor growth, and improved survival compared with vaccines based on unmodified ENO1 sequences. Importantly, the therapeutic effect was markedly enhanced when vaccination was combined with PD-1 blockade, leading to tumor eradication in a substantial proportion of treated animals. These findings provide direct in vivo evidence that cancer-associated citrullination of ENO1 can be exploited to design vaccines with enhanced immunogenicity and combinatorial potential with immune checkpoint inhibition [93].

Notably, ENO1 has also been proposed as a candidate target for cancer immunoprevention, as ENO1-directed vaccination in preclinical pancreatic cancer models was effective when administered at premalignant stages, delaying tumor onset and reshaping early immunosuppressive microenvironments [94]. Taken together, these findings further support the rationale for ENO1-based vaccination and its integration with complementary therapeutic approaches.

Finally, pharmacologic targeting of ENO1 has shown immunomodulatory efficacy in non-oncologic inflammatory models. The natural compound paeoniflorin directly binds ENO1, reduces its enzymatic activity and glycolytic flux, and limits pro-inflammatory macrophage polarization, providing proof-of-principle that ENO1 is pharmacologically druggable as a metabolic–immune node [95].

### 6.3. Translational Challenges and Future Perspectives

Despite these promising findings, several translational challenges remain before ENO1-directed strategies can be clinically implemented. Most available evidence derives from genetically engineered or syngeneic murine models, which only partially recapitulate the complexity and immune heterogeneity of human tumors. In addition, no clinical studies have yet evaluated ENO1-targeted vaccination or antibody-based strategies in cancer patients. Another important challenge concerns safety, given the widespread physiological expression of ENO1 as a ubiquitous housekeeping glycolytic enzyme. Although the available preclinical evidence is encouraging, safety evaluation has not been addressed uniformly across studies. Full-length ENO1 DNA vaccination was associated with selective sparing of normal cells expressing lower endogenous ENO1 levels, and histological analyses included several organs [86]. More comprehensive safety assessment was reported for the citrullinated ENO1 peptide vaccine, in which histopathological examination of multiple organs revealed no evidence of treatment-related toxicity in mice [93]. By contrast, although the humanized anti-ENO1 antibody HuL001 demonstrated promising antitumor activity and enhanced the efficacy of radiotherapy in preclinical models, detailed systemic toxicity analyses have not yet been reported [35]. Overall, these findings suggest that the available preclinical evidence has not raised major safety concerns to date, while also highlighting the need for more standardized and comprehensive safety evaluation before clinical translation. Future studies will therefore need to clarify the relative contribution of ENO1-dependent pathways across distinct tumor contexts, establish their long-term safety, and determine which patient subsets are most likely to benefit from ENO1-directed therapeutic approaches.

## 7. Conclusions

The evidence reviewed here positions ENO1 at a functional intersection where metabolic adaptation, antigen exposure, and immune regulation converge. Together, these properties make ENO1 both a mechanistically informative model for studying tumor–immune crosstalk and a rational target for therapeutic intervention.

Beyond its canonical role as a glycolytic enzyme, ENO1 can become immunologically relevant through non-canonical mechanisms including increased expression, stress-associated surface exposure, and post-translational modification. These processes transform a ubiquitous housekeeping protein into a tumor-associated antigen capable of eliciting both humoral and T-cell responses across multiple malignancies.

At the same time, ENO1 participates in immunometabolic programs that shape the tumor immune microenvironment. Depending on cellular, metabolic, and tumor-specific context, ENO1 can promote antitumor immune recognition or contribute to immune suppression through coordinated effects on myeloid remodeling, macrophage polarization, suppressive immune-cell trafficking, and checkpoint-associated pathways. Although the relative contribution of these mechanisms likely varies across tumor types and microenvironmental contexts, the available evidence collectively supports a context-dependent role for ENO1 in regulating tumor–immune interactions.

These features provide a strong rationale for exploiting ENO1 in cancer immunotherapy. Preclinical studies demonstrate that ENO1-directed vaccination, antibody-based targeting, and rational combination strategies can generate productive antitumor immune responses. However, several important questions remain unresolved. The relative contribution of ENO1-dependent immunoregulatory mechanisms across different tumor types, disease stages, and microenvironmental contexts remains incompletely understood, and the factors determining whether ENO1 predominantly promotes antitumor immunity or immune suppression require further clarification. Addressing these issues will require the identification of predictive biomarkers capable of stratifying patients according to ENO1-associated immune states, together with validation of these mechanisms in clinically representative models and early clinical studies. Ultimately, integrating ENO1-targeted strategies with established immunotherapies may enable more effective and context-dependent approaches to cancer immunotherapy.

## Figures and Tables

**Table 1:** Major post-translational modifications of ENO1 discussed in this review and their relevance to cancer biology and immune regulation.

Post-Translational Modification	Biological Significance	Immunological Relevance	Discussed in
Citrullination	Converts arginine residues into citrulline, altering ENO1 structure and generating modified peptide epitopes	Direct immunogenic effect. Enhances MHC class II presentation and CD4^+^ T-cell recognition; exploited in ENO1-based vaccination strategies	Section 3.1 and Section 6.2.2
Phosphorylation	Generates phosphorylated ENO1 species containing immunologically distinct phosphopeptides	Direct immunogenic effect. Promotes HLA-restricted T-cell recognition and contributes to inter-individual variability in immune responses	Section 3.2
Lysine acetylation	Regulates the RNA-binding activity of ENO1, linking the protein to riboregulation and post-transcriptional control	No direct link to ENO1 immunogenicity is discussed in the evidence reviewed here	Section 1
Symmetric dimethylation	Promotes PRMT5-dependent ENO1 translocation to the plasma membrane	Indirect immunoregulatory effect. Modulates ENO1 immune-related functions through increased surface exposure rather than neo-epitope generation	Section 2.2
Persulfidation	Enhances ENO1-dependent regulatory T-cell activation	Indirect immunoregulatory effect. Contributes to Treg-mediated immune suppression within the tumor microenvironment	Section 5.1
O-GlcNAcylation	Weakens the ENO1–PD-L1 interaction, reducing STUB1-mediated PD-L1 ubiquitination and degradation	Indirect immunoregulatory effect. Promotes PD-L1 stabilization and tumor immune evasion	Section 5.3.1
Ubiquitination	Regulates ENO1 protein stability through proteasomal degradation	Indirect immunoregulatory effect. Controls ENO1 abundance, thereby influencing tumor metabolism and immune-related functions	Section 5.3.2 and Section 5.4

## Data Availability

No new data were created or analyzed in this study. Data sharing is not applicable to this article.
